# The effectiveness of JU:MP a whole system approach to improve physical activity of children aged 5 to 11 years living in multi-ethnic and socio-economically deprived communities: a non-randomised controlled trial

**DOI:** 10.1186/s12889-025-25772-9

**Published:** 2025-12-07

**Authors:** Sally E. Barber, Daniel D. Bingham, Nathan P. Dawkins, Zoe Helme, Jennifer Hall, Amanda Seims, Gillian Santorelli, John Wright, Rosie RC McEachan, Jan Burkhardt, Andy Daly-Smith

**Affiliations:** 1 Bradford Institute for Health Research, Bradford Teaching Hospitals NHS Foundation Trust, Bradford Royal Infirmary, Duckworth Lane, Bradford, BD9 6RJ UK; 2 Institute for Health and Social Care, University of Bradford, Richmond Road, Bradford, BD7 1DP UK

**Keywords:** Physical activity, Whole system, Children, Inequalities, Non-randomised controlled trial

## Abstract

**Background:**

Whole system approaches to public health challenges such as low physical activity levels have the potential to create sustained behaviour change at a population level and tackle health inequalities. However, there is currently little evidence of the nature or effectiveness of adopting whole system approaches. This study evaluated whether a whole system physical activity intervention (JU:MP), was effective at improving accelerometry measured physical activity in five- to eleven-year-olds.

**Methods:**

A non-randomised controlled trial with two-arms (JU:MP intervention and control), was conducted in multi-ethnic and socioeconomically deprived areas of Bradford, UK with data collected at baseline and 24-months follow-up. Habitual physical activity was measured via accelerometry. Mixed effects regression models identified group differences at 24 months. The primary outcome was moderate-to-vigorous intensity physical activity (MVPA). Secondary outcomes included: accelerometery measured - sedentary time (ST), counts per minute (CPM); BMI z-score, waist circumference, and children’s social, emotional and behavioural health, and quality-of-life via parental and teacher completed questionnaires. An exploratory analysis compared intervention effects between sub-groups.

**Results:**

1,453 children were recruited. 330 children with valid wear-time at baseline and 24-months (JU:MP group *n* = 175, control group *n* = 155) were included in the final analysis of physical activity outcomes. The JU:MP group improved levels of MVPA (+ 4.99 min/day, (CI = 1.01, 8.96), standardised mean difference (SMD) = 0.29), ST ( -8.69 min/day, CI = -16.76, -0.61), SMD = -0.20) and CPM (+ 32.72, CI = 5.93, 59.53, SMD = 0.28) compared to controls. There were minor differences between groups in all secondary outcomes, favouring the JU:MP group. Exploratory sub-group analysis revealed that MVPA improved for boys (+ 7.34 min/days, CI = 0.70, 13.99, SMD = 0.36) and South Asian heritage children (+ 7.20 min/day, CI = 1.67, 12.72, SMD = 0.52) in the JU:MP group compared to the control group.

**Conclusion:**

This study provides evidence that a whole system, community-based intervention can improve physical activity levels in primary school-aged children, particularly among boys and South Asian children, in deprived and ethnically diverse settings. The findings suggest that whole systems approaches may be effective in mitigating age-related declines in activity and addressing inequalities at scale.

**Trial registration:**

This study was retrospectively registered with the ISRCTN registry (ISRCTN14332797) on 17/02/2022. Available at: https://www.isrctn.com/ISRCTN14332797.

**Supplementary Information:**

The online version contains supplementary material available at 10.1186/s12889-025-25772-9.

## Background

Improving children’s levels of physical activity is a high priority for public health [1, 2]. Most (~ 70%) of the children in the UK do not meet the Chief Medical Officers guidelines of an average of 60 min moderate-to-vigorous intensity physical activity (MVPA) a day [3]. Self-report data from Sport England’s Active Lives Children and Young People Survey indicate that 52.2% of 5–16-year-olds do not meet the guideline, illustrating methodological differences but confirming that the majority of children remain inactive [4]. There are also inequalities in activity levels, with children from black and Asian ethnic minority backgrounds and those from the least affluent families less active than other ethnicity and affluence groups [4]. Girls are also less active than boys across all ages in childhood [4]. Undertaking sufficient daily physical activity and reducing sedentary time is beneficial for children’s physical, social and emotional health, their growth and development and prevents the early onset of diseases [5]. Physical activity behaviours typically decline across childhood and adolescence from age 6–7 through to age 14–15 and they track from childhood, through adolescence and into adulthood [6]. Establishing adequate physical activity behaviours and mitigating this decline can have life-long benefits for health and wellbeing. In addition to the health-related consequences of physical inactivity, it is estimated the economic cost of inactivity in the UK is £7.4 billion each year [2], which emphasises the urgent need to address this issue.

The physical activity and movement behaviours of populations are influenced by complex political, environmental and social systems rather than just an individual’s ‘intention’ to be active [1]. Whole system approaches that intervene in these complex systems to improve population level physical activity are advocated for by the World Health Organisation and the International Society of Physical Activity and Health [1, 7]. Authentic whole-system approaches draw on complexity science, which is the study of systems composed of diverse, interacting agents whose local behaviours and feedback loops give rise to unpredictable, emergent outcomes; and complex adaptive systems, which self‐organise and adapt over time in response to changing condition [8]. They should contain, [1] heterogeneous interacting elements, [2] an emergent effect that is different from the effects of the individual elements, and [3] persisting effects over time that adapt to changing circumstances [9]. Multi-faceted interacting interventions which work at various ‘levels’ of a whole system (e.g. individuals, communities, organisations, environment and policy and strategy) [9, 10], have the potential to lead to sustained behaviour change at a population level [9, 10], and may help to reduce health inequalities. Despite system approaches to physical activity being increasingly advocated for, evidence of their operationalisation and effectiveness is sparse [11, 12].

A scoping review by Nau, Bauman [11] identified seven studies which focused on system approaches for adult physical activity [11]. There were no whole system approaches to improve children’s physical activity per se, but four designed for childhood obesity prevention, which included some elements targeting physical activity behaviours. Nau et al. concluded that this field of research is at an early stage of development, with most literature reporting descriptives of approaches and providing no further complex analyses [11]. A further systematic review by Koorts et al. found eight interventions with high or moderate use of systems approaches to drive impact at scale to tackle non-communicable diseases, which included a physical activity component [12]. Only one targeted children [13]; it took a whole-of-school approach (e.g. a school system approach) rather than targeting multiple settings across a whole ‘local’ system (e.g. children, families, the wider community, schools, community organisations, faith settings, environments including green spaces and active travel infrastructure, local government policy and strategy) and was deemed as having moderate use of a systems approach.

Since 2018, Sport England (a non-departmental public body of the UK government, which funds and supports people and communities across England to be active) has funded twelve localities across England to pilot whole system, place-based approaches to reduce physical inactivity and health inequalities [14]. One of these localities was Bradford, where the approach, called JU: MP, aimed to improve physical activity in children aged five to fourteen years living in some of the most deprived areas of England, with a focus on tackling physical activity inequalities for girls and for ethnic minority groups [15]. Several other UK localities have been supported by Sport England to take a place-based approach, including those in Sheffield, Liverpool, and Greater Manchester in the UK. They have been evaluated primarily through qualitative and mixed-methods studies, highlighting enhanced partnership working, community engagement, and system learning [14, 16]. Reported changes in self-reported activity within these areas have been modest and variable, and to date no evaluations of this place-based working has used device-measured outcomes at scale. To our knowledge, JU: MP represents the first controlled trial to assess the effectiveness of whole system, place-based working on objectively measured MVPA in children, addressing a critical gap in the evidence base.

The primary aim of this study was to explore whether JU: MP; a whole systems community-based physical activity approach delivered at scale, was effective at improving MVPA among children aged five to eleven years old. Secondary aims were to determine the effectiveness of the intervention on impacting broader measures of physical behaviours, adiposity, social emotional and behavioural health, and quality of life.

## Methods

### The JU: MP intervention

**Fig. 1:**
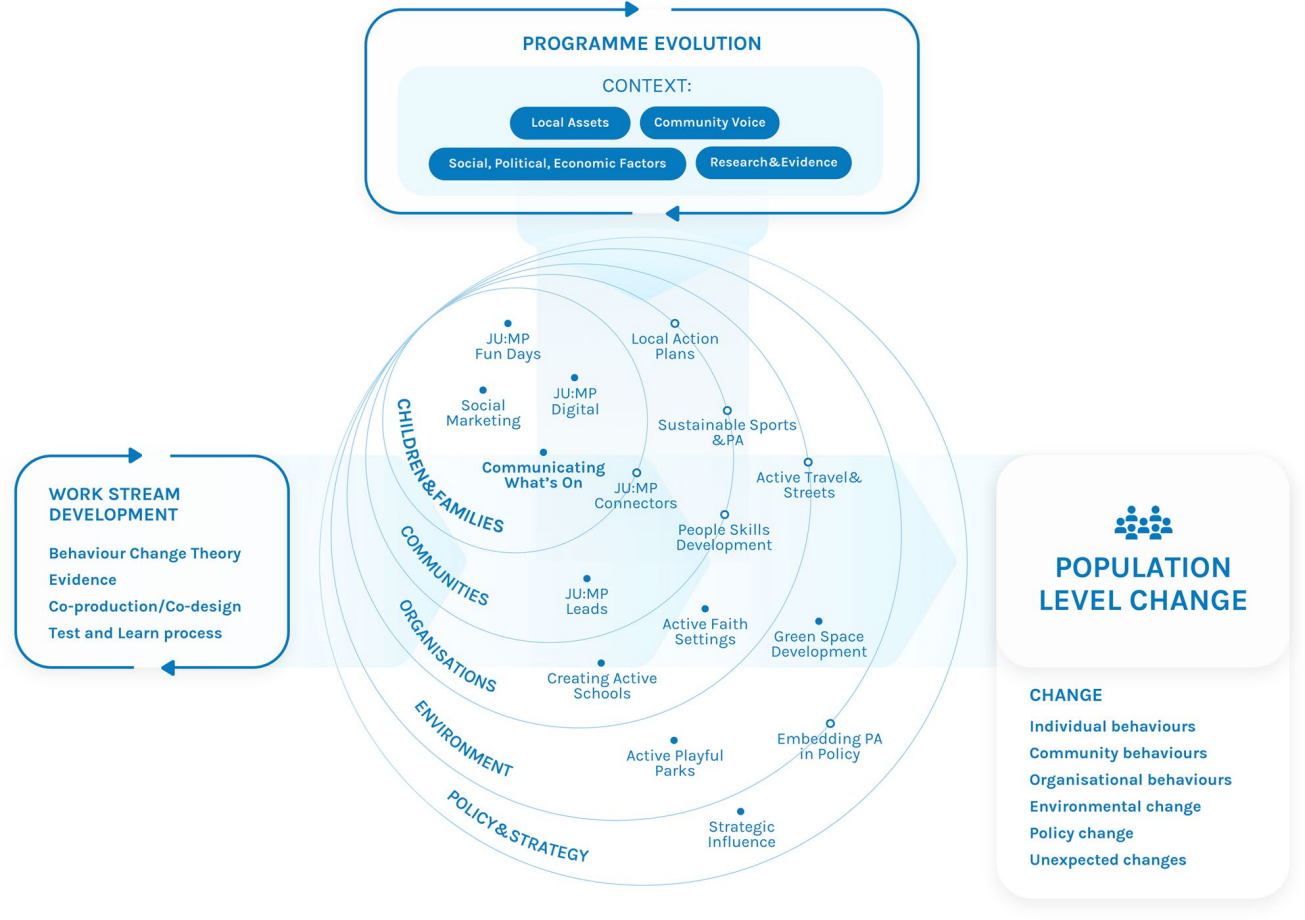
JU: MP Conceptual Model illustrating how multiple interconnected work streams and theoretical foundations combine to promote population-level change in children’s physical activity across the Bradford local system

The JU: MP intervention has been described in greater length and detail in previous published protocols[17, 19]. For the purposes of this paper a summary is provided. The JU: MP intervention is grounded in theory and evidence, integrating complex adaptive systems thinking, the socio-ecological model, and COM-B behaviour change theory, alongside insights from the Determinants of diet and physical activity (DEDIPAC) reviews [20–23], local evidence (e.g. a community priority-setting exercise in Bradford which identified safe play spaces and culturally appropriate activity opportunities as priority concerns [24]; and was co-designed with community stakeholders. It operated across five interconnected components of the local system (policy and strategy, built environment, community networks, organisational settings, and children and families), ensuring that activities in each domain reinforced one another. Sixteen work streams that sat within at least one element of the ‘local system’ (and often multiple) were developed and implemented in eight geographically defined neighbourhoods across the north of Bradford city reaching 30,000 children. The intervention aimed to increase capability, opportunity and motivations for physical activity in the targeted communities. This included (but was not limited to) creating safer more appealing play spaces, increasing opportunities for active transport, creating a culture for physical activity in schools, faith settings, and community organisations, and educating and motivating families and community members through physical activity social marketing campaigns. The JU: MP Conceptual Model (Fig. 1) illustrates how JU: MP integrated theoretical frameworks (complex adaptive systems, socio-ecological, and COM-B) within an iterative ‘test-and-learn’ process, allowing activities across sectors and settings to align and adapt dynamically. The model highlights how change was pursued simultaneously at multiple levels of the system, individual, community, organisational, environmental, and policy, through feedback loops, co-production, and shared learning. This systems-based design aimed to enable sustainable change by embedding physical activity within everyday community life, practice, and infrastructure. The different intervention elements interacted with each other via various mechanisms including staff whose role it was to connect across the system and local neighbourhood action groups whereby partners from across the system regularly came together to design and deliver a local action plan. An iterative learning approach was taken to understand what worked (and did not work) for who, how and why; this supported the continuous development of JU: MP and allowed it to adapt to the changing social, political and economic context. In each neighbourhood JU: MP had an intensive 24-month delivery phase followed by a 6 month embed and sustain phase in which financial support for activities tailed off and activities were supported to self-sustain. The JU: MP intervention was piloted in three neighbourhoods between 2019 and 2021, after this the intervention was delivered in a further five neighbourhoods in 2021–2024. Three of the 2021–2024 neighbourhoods were used to conduct the effectiveness study, which was designed to evaluate JU: MP’s effect on device-measured MVPA in children and represents one strand of a wider mixed-methods evaluation that includes process, economic and participatory studies which are being published at a later date [17, 19]. The resources available to conduct an accelerometery based outcome study, only permitted the inclusion of only three intervention neighbourhoods and their matched controls in a longitudinal effectiveness trial. These three areas were chosen by the research team, before any implementation occurred, to ensure representation of the main ethnic groups in Bradford, one predominantly White British, one predominantly British South Asian, and one mixed-ethnicity; while all five neighbourhoods shared comparable socio-economic deprivation.

### Study design

The effectiveness of the JU: MP intervention was assessed using a two-arm (JU: MP intervention and control), non-blinded, non-equivalent group before and after study design; outlined in our published protocol [18](ISRCTN14332797). A controlled evaluation was chosen over a randomised control trial because JU: MP was rolled out in a real-world, phased manner across neighbourhoods, and random allocation was not feasible given pre-existing funding timelines and ethical commitments to communities. Instead, three intervention neighbourhoods were compared with matched control areas to balance key demographic and environmental factors. This pragmatic, natural-experiment approach allowed us to evaluate JU: MP alongside ongoing implementation while preserving community trust. We collected data at three waves (baseline, 24 months at the end of intensive delivery, and 36 months follow-up), with the current study focusing on baseline and 24-month measures. The decision to report the baseline and 24-month data was made to provide timely evidence on JU: MP’s effects following its intensive delivery phase. JU: MP was designed as a phased, whole-system intervention comprising three overlapping stages: (1) development, involving community engagement, co-production, and pilot testing; (2) implementation, representing a 24-month period of intensive multi-level delivery across schools, families, and community settings; and (3) maintenance, focused on sustainability, and system embedding from 24 to 36 months. The 24-month follow-up therefore marks the end of the intensive implementation phase and the primary outcome period. Reporting the 24-month outcomes separately was necessary both to meet Sport England’s requirement for interim results and to respond to community partners’ need for early feedback to inform ongoing planning, decision-making, and resource allocation. The 36-month analyses will be presented in a subsequent publication, focusing on the maintenance and sustainability phase and the longer-term system-level impacts of JU: MP (see Fig. 1 for the participant flow diagram). All procedures were approved by the University of Bradford Research Ethics Committee (HSR Panel; ref E891, July 2021).

### Patient and public involvement and engagement (PPIE)

We followed the NIHR INVOLVE principles to ensure systematic, transparent engagement of communities throughout the study [25]. During conception of the study design and methodology we consulted with the Born in Bradford Parental Involvement group and the Born in Bradford Young Ambassadors group (children were aged 9–12 years old) to explore measurement tools and protocols that would be feasible and acceptable for children and parents. We attended a Bradford Schools Linking Network meeting as part of the Centre for Applied Education Research [26] and discussed with teachers and school senior leadership staff how the study could be delivered in schools with minimal disruption to the school day. After the baseline data collection, we held some small group discussions with children who had taken part in the data collection to understand what would improve their experience of participation for the 24-month follow-up, because of this we employed privacy screens to measure waist circumference during follow-up measures. To engage with study participants throughout the study we created and shared child-friendly animations of the different aspects of the study (e.g. what happened during data collection, what happened to their data after it had been collected and a summary of the findings of the study).

### Sampling and recruitment of school and participants

The JU: MP intervention group was recruited from three neighbourhoods selected to ensure that children with White British ethnicity and children from ethnic minority groups were represented in the trial. Neighbourhoods were originally identified through a mapping exercise conducted by the JU: MP implementation team who used ONS ward-level population and ethnicity data alongside local school census figures, with final feasibility confirmation conducted in collaboration with community stakeholders. Control neighbourhoods were selected from within Yorkshire and matched to the JU: MP intervention neighbourhoods (as according to the protocol) to ensure socio-demographic comparability. The matching was based on school-level Index of Multiple Deprivation (IMD) [27](maximum 1 decile difference), free school meal eligibility (maximum 10% difference), and predominant ethnic group composition (maximum 15% difference). Within the selected areas, government-funded primary schools were invited to participate in the study, with incentives offered for participation, which was a sum of £200 per data collection, which schools agreed would not be spent on physical activity or sport resources. All children in Years one, two, and three (ages five to eight years) at recruited schools were invited to participate. Parents/carers provided informed consent for their child to participate before data collection. On the day of data collection, each child provided verbal assent, with appropriate support provided for those children with special educational needs/disabilities (e.g. a school member of staff known to the child, such as a teacher assistant or teacher was present during all contact with children with special needs, to advocate for the child and inform the researchers if they felt assent had been withdrawn). All children received a gift “goodie bag” (i.e. colourful tote bag with pencils, note pads, flower seeds, and an age-appropriate reading book) at each data collection time-point.

At baseline, schools provided demographic data including child date of birth, biological sex, ethnicity, home postcode (for IMD calculation), child’s disability or special educational needs, and receipt of government-funded free school meals for each participant.

### Outcome measures

At each time-point the following measures were collected for children unless otherwise stated: accelerometer assessed physical activity, anthropometric data, social emotional and behavioural health (teacher rated), and questionnaire assessed physical activity, sedentary behaviour, sleep, and quality of life (parent/carer rated).

#### Accelerometer assessed physical activity

Physical activity and sedentary time were measured using waist-worn ActiGraph accelerometers placed on the participant’s right hip, MVPA was the primary outcome variable. Participants were asked to wear the accelerometer for seven consecutive days (24-hours a day). The ActiGraph data was processed using ActiLife v6 (ActiGraph) and downloaded in 60-second epoch files. Sleep was identified using the validated sleep period–detection algorithms [28, 29], and removed from the physical activity analysis. Physical activity intensities were classified using the Evenson cut points [30]. To ensure consistency and increase validity, data was reprocessed using software Actilife v6, to generate 15-second epoch data to align with Evenson’s protocol [30, 31]. From this, data were categorised to establish the following outcome variables: sedentary time (ST), light physical activity (LPA), moderate-to-vigorous physical activity (MVPA), total physical activity (TPA), and average daily counts per minute (CPM). Non-wear time was defined as 20 min of consecutive zeros and was removed from the data. The wear-time criteria used was 600 min of wear to establish a valid day. Participants required three days of wear time, including at least one weekend day, at both baseline and follow-up, to be included in the analysis. This wear-time criterion was calculated using published local Bradford data and has an estimated intraclass correlation of 0.75 [32, 33].

#### Anthropometric data

Anthropometric measurements were collected during data collection visits at each time point, with each measure repeated three times. Participants were requested to remove shoes and jumpers for each anthropometric measurement. Height (cm) and mass (kg) were measured, using a portable stadiometer (Seca 213, Hamburg, Germany and digital scales (Seca 875, Hamburg, Germany), to the nearest 0.1 cm and 0.1 kg respectively. BMI was calculated and converted to a BMI percentile and z-score based on UK reference data [34]. Waist circumference (cm) was measured to the nearest 0.1 cm at the narrowest point between the bottom rib and the iliac crest, in the midaxillary plane, using an ergonomic circumference tape (Seca 201 Hamburg, Germany).

#### Children’s social emotional and behavioural health (teacher rated)

Social, emotional and behavioural health was assessed by class teachers using the paper unedited version of the strengths and difficulties questionnaire (SDQ)[35]. The SDQ contains 25 items, grouped into 5 subscales: prosocial behaviour (e.g., acts of kindness, sharing, and helping others), emotional problems (e.g., feelings of anxiety, sadness, or worry), behavioural problems (e.g., rule-breaking, temper tantrums, or disobedience), peer problems (e.g., difficulties making or keeping friends, being bullied), and hyperactivity/inattention (e.g., restlessness, fidgeting, or difficulty concentrating). The SDQ teacher version has been found to have acceptable validity for evaluating psychosocial functioning in children [36],and has been widely used within public health research within the UK and Bradford [32, 33].

#### Questionnaire assessed physical activity, sedentary behaviour, sleep, and quality of life (parent/carer rated)

Parents/carers completed a questionnaire to assess their child’s physical activity, sedentary behaviour, sleep and quality of life. The questionnaire consisted of six sections which covered: (1) personal information; (2) The Youth Activity Profile; (3) sleep duration; (4) parent-reported physical activity in specific settings; (5) parental practices; and (6) quality of life. Section one, personal information included: school class, teacher’s name, age and the relationship to the person completing the questionnaire. Section two comprised the Youth Activity Profile (YAP), a 15-item parent‐proxy measure with a 10‐item physical activity subscale (items scored 0 “none” to 5 “7 days,” range 0–50; Cronbach’s α = 0.71 at baseline and follow-up) and a 5-item sedentary behaviour subscale (same 0–5 scoring, range 0–25; α = 0.62 at both time points)[37]. Section three recorded weekdays’ and weekends’ bedtimes and wake‐times, from which average sleep duration was calculated [38]. Section four assessed the children’s parent-reported physical behaviours. This section included questions on the frequency and locations where children were physically active for at least 10 min over the last 7 days; and how frequently children were physically active in parks and green spaces. In addition, if the child was of Muslim faith, parents were asked to complete a subsection including questions on the frequency of attendance at a Mosque or Madrasa; the duration of stay; and whether there was any active travel to or from the Mosque or Madrasa. Section five included questions focussed on physical behaviours and neighbourhood walkability. These questions were taken from previously validated questionnaires [39, 40]. Finally, section six measured health-related quality of life via the EQ-5D-Y proxy (5 three-level items; utility 0–1; α = 0.64 at baseline, 0.66 at follow-up) and the PedsQL generic core (23 items across physical, emotional, social, and school functioning; 5-point Likert 0 “never” to 4 “almost always,” transformed to 0–100; α = 0.92 at baseline and 0.92 at follow-up)[41–43].

### Statistical analysis

Power calculations are detailed in the published protocol [23]. Briefly, the calculation accounted for 6 clusters (3 JU: MP intervention and 3 control neighbourhoods), with a 5% two-sided alpha. Based on pilot data from 564 children across 12 primary schools situated within three JU: MP neighbourhoods, an assumed control mean of 53.7 min of MVPA per day, a standard deviation of 19.7, and an intracluster correlation coefficient (ICC) of 0.007 (rounded to 0.01) were used. This ICC was empirically derived from the JU: MP pilot study using accelerometer-measured MVPA and reflects clustering at the school level nested within neighbourhoods. More recent pooled UK data suggest somewhat higher ICCs (0.04–0.08) for accelerometer-measured MVPA in primary schools [44], but the pilot-derived value was considered appropriate at the time of design given JU: MP’s neighbourhood-based implementation and evaluation structure. To detect a 10-minute difference in the primary outcome of average daily MVPA, with 80% power at 24-month follow-up, accounting for 30% baseline accelerometer noncompliance and 50% follow-up data attrition; a minimum sample of 1,200 children (600 per condition group) was recommended, leaving a minimum final sample of 350 children (175 per condition group). Mixed effects regression models assessed JU: MP intervention effects on physical activity and sedentary outcomes, accounting for individual and group-level factors. The primary outcome was the difference between control and JUMP groups average daily MVPA at 24 months follow-up, while secondary outcomes included differences between groups average daily ST, LPA, TPA, and CPM. Random intercepts were included for clustering effects at the school and neighbourhoods levels [45]. Given the relatively small final sample of participants with both valid baseline and follow-up data (*n* = 330) and the natural skewness of accelerometer data [46, 47]; bootstrapping with 10,000 replications was used to enhance robustness, providing robust standard errors [48] and confidence intervals [49]. Bootstrapping has been used in physical activity research to improve estimate stability in cases of skewed data or limited sample sizes, as recommended in general methodological literature [48, 49] and applied in previous physical activity data analyses [48, 50–52]. Standardised mean differences (SMD) measured effect sizes and were interpreted as small (0.2 to 0.5), medium (0.5 to 0.8), or large (≥ 0.8) [53]. Adjusted means for the JU: MP intervention and control groups were calculated using post-hoc predictive margins. All models were adjusted for covariates, including sex, ethnicity, baseline values, wear time, BMI z-score, age, receipt of free school meals, school, and neighbourhood. Analyses followed a completers analysis approach, incorporating all participants with valid baseline and follow-up data to maintain group assignment [54]. To assess potential bias arising from missing accelerometer data, inverse-probability-of-attrition weighting (IPW) was applied to the primary outcome (mean daily MVPA)[55]. Stabilised weights were derived from a logistic regression predicting the likelihood of providing valid follow-up accelerometer data based on baseline covariates with minimal missingness (sex, ethnicity, free school meal eligibility, and trial arm). IPW methods provide a principled approach for reducing attrition bias under a missing-at-random assumption [56, 57]. The distribution of stabilised weights was narrow and centred around 1.0 (mean 1.02, SD 0.14, range 0.84–1.32; Supplementary Table S2), indicating no evidence of extreme or unstable values. An augmented IPW model additionally included baseline MVPA and self-reported physical activity (YAP). IPW provides a pragmatic approach to assess whether selective attrition biased the completers’ estimates. All analyses were conducted in STATA v.17, and statistical significance was set at *p* < 0.05.

## Results

### Recruitment and control identification

Figure 1 outlines the flow chart of schools and participants. Between June and December 2021, 95 primary schools across the Yorkshire region in England were approached; 17 out of the 19 intervention schools approached (89%), and 20 out of the 79 control schools approached (26.3%) agreed to participate. Control neighbourhoods were closely matched on the number of eligible pupils and the ethnicity of pupils (less than 5% difference between pupils who were classed as White British and South Asian). However, control neighbourhoods had a lower proportion of children eligible for free school meals (30.7% v 26.6%), and a higher median school IMD decile score (1 v 2) (refer to Supplementary Table S1, for details regarding the school and neighbourhood characteristic).

Of 4,408 eligible children (school year group 1 to 3), 1,453 parents/carers provided consent (34.2% JU: MP intervention, 31.7% control). A total of 316 children (21.7%) dropped out of the study at 24 months follow-up (T1), reasons being, school withdrawal (1 JU: MP school, 2 control schools), leading to *n* = 99 (6.8%), dropping out. A further 217 (14.9%) children dropped out due to absence/illness during data collection. For accelerometry, 800 (55.1%) children met the wear-time criteria at baseline, 505 (34.8%) at 24 months, leaving a final sample of 330 (22.7%) which met the wear-time criteria at both time points and were included in the primary outcome analysis and analysis of other physical activity variables. For other secondary outcomes a total of 1,103 (75.9%) children had anthropometric data, 702 (48.3%) parent questionnaire data and 716 (49.3%) teacher questionnaires at both baseline and follow-up (Fig. 1).

### Demographics

**Fig. 2:**
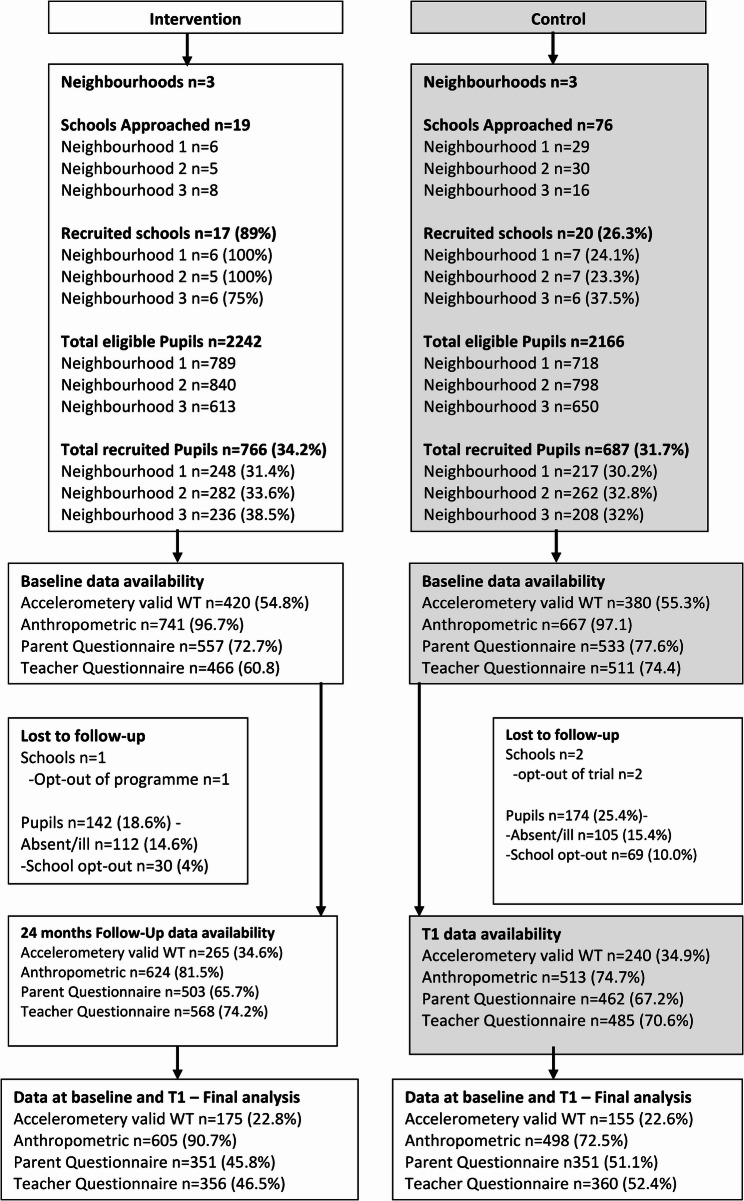
Flowchart of participant recruitment, retention, and inclusion in the final analysis

Baseline demographic characteristics are presented in Table 1. Intervention (*n* = 766) and control (*n* = 687) groups showed no differences in sex but differed in ethnicity and socio-economic indicators at baseline (*n* = 1,453). The JU: MP intervention group had fewer white British participants (38.9% vs. 44.4%) and higher free school meal eligibility (30.6% vs. 25.9%). Deprivation was greater in the JU: MP group, with 72.2% of children in the most deprived quintile versus 58.1% in controls. Physical activity outcomes showed minor differences, with the JU: MP group exhibiting higher light physical activity (LPA) and total physical activity. Sedentary time and MVPA did not differ between groups. Descriptive analysis compared the final analysis sample (*n* = 330) with participants excluded from the main analysis due to not meeting accelerometer wear time criteria (*n* = 1,123), see Table 2. Both groups had a similar male-to-female ratio and comparable ethnic composition. However, socioeconomic differences were observed; fewer participants in the final sample were eligible for free school meals, and fewer were from the most deprived quintile. More final sample participants resided in Neighbourhood 3, age and BMI Z-scores were similar across groups (Fig. 2).

**Table 1:** Demographic descriptive statistics of recruited sample and baseline accelerometer data

Total Sample: *n* = 1453	Intervention *n* = 766 (52.7%)		Control *n* = 687 (47.3%)		*P*-Value
	*N*/Median	%/IQR	*N*/Median	%/IQR	
Sex					
Female	411	53.66	357	51.97	0.572
Male	355	46.34	330	48.03	
Ethnicity					
White British	298	38.9	305	44.4	0.04
South Asian	342	44.65	295	42.94	
Other	126	16.45	87	12.66	
Special Educational Needs or disability					
Yes	78	10.18	73	10.63	0.782
No	688	89.82	614	89.37	
Health condition					
Yes	128	16.71	570	17.03	0.871
No	638	83.29	117	82.97	
Parent/carer Consent					
Mother	598	78.07	549	80.03	0.162
Father	151	19.71	117	17.06	
Other	17	2.22	21	2.91	
Free School Dinners					
Yes	234	30.63	178	25.91	0.047
No	530	69.37	509	74.09	
Index of Multiple Deprivation (Quintile)					
1	553	72.19	399	58.08	0.000
2	171	22.32	222	32.31	
3	20	2.61	29	4.22	
4	16	2.09	31	4.51	
5	6	0.78	6	0.87	
Neighbourhoods					
1	236	30.81	208	30.28	0.877
2	282	36.81	262	38.14	
3	248	32.38	217	31.59	
Age years	6.78	6.07, 7.46	7.05	6.27, 7.73	0.000
Body Mass Index Z-score	0.382	−0.442, 0.382	0.229	−0.549, 1.202	0.0414
MVPA average daily minutes*	66.37	23.25	66.68	22.06	0.8434
Sedentary Time average daily minutes*	387.21	58.98	392.98	46.15	0.1262
Light PA average daily minutes*	327.63	53.59	315.18	43.59	0.0004
Total PA average daily minutes*	393.99	64.44	381.86	53.95	0.0042
Wear time average daily minutes*	781.20	50.83	774.85	49.33	0.0737
Counts Per minute (CPM)*	646.58	161.90	645.42	143.01	0.9149

*Data for participants with valid data (800 participants at baseline)

Index of Multiple Deprivation (Quintile): 1 = 20% most deprived postcodes in England, 5 = 20% most affluent postcodes in England

*PA *Physical Activity

*MVPA *moderate-to-vigorous physical activity

Health condition is a state of illness, injury or diseases that was documented within the schools pupil records

**Table 2:** Demographic descriptive statistics of final analysis sample

	Sample not included in main analysis *n* = 1123		Main final Analysis Sample *n* = 330		*P*-Value
	*N*/Median	%/IQR	*N*/Median	%/IQR	
Sex					
Female	603	53.7	165	50	0.237
Male	520	46.3	165	50	
Ethnicity					
White British	466	41.5	137	41.52	0.681
South Asian	497	44.26	140	42.42	
Other	160	14.25	53	16.06	
Special Educational Needs or disability					
Yes	126	11.22	25	7.58	0.057
No	997	88.78	305	92.42	
Health condition					
Yes	186	16.56	59	17.88	0.575
No	937	83.44	271	82.12	
Parent/carer Consent					
Mother	872	77.72	275	83.33	0.377
Father	216	19.25	52	15.76	
Other	35	3.03	3	0.91	
Free School Dinners					
No	779	69.49	260	78.79	0.001
Yes	342	30.51	70	21.21	
Index of Multiple Deprivation (Quintile)					
1	759	67.59	193	58.48	0.002
2	276	24.58	117	35.45	
3	42	3.74	7	2.12	
4	37	3.29	10	3.03	
5	9	0.8	3	0.91	
Neighbourhoods					
1	358	31.88	86	26.06	0.001
2	433	38.56	111	33.64	
3	332	29.56	133	40.3	
Age years	6.92	6.18, 7.61	6.93	6.10, 7.75	0.8923
Body Mass Index Z-score	0.306	−0.50, 1.31	0.27	−0.57, 1.23	0.0414

Index of Multiple Deprivation (Quintile): 1 = 20% most deprived postcodes in England, 5 = 20% most affluent postcodes in England

*PA *Physical Activity

*MVPA *moderate-to-vigorous physical activity

Health condition is a state of illness, injury or diseases that was documented within the schools pupil records

### Accelerometry outcomes

Physical activity results are reported in Table 3. There was evidence of a meaningful effect of the JU: MP intervention on the primary outcome, average daily habitual MVPA, with an adjusted mean difference of + 4.99 min/day in the JU: MP group compared to the control group at 24 months follow-up. The effect was stronger on weekdays, with a difference of + 5.77 min/day (SMD = 0.35) compared to a difference of + 4.18 min/day on weekends (SMD = 0.15). Secondary accelerometer outcomes showed meaningful differences in average daily ST with a difference of −8.69 min/day (SMD = −0.20) between JU: MP and control group at 24 months follow-up and a larger difference observed on weekends (−21.47 min/day, SMD = −0.40). The difference in average daily light physical activity (LPA) between JU: MP and control groups at 24 months was only different on weekend days + 11.75 min/day (SMD = 0.29). Additionally, counts per minute (CPM), a measure of total physical activity, improved by 32.72 CPM (SMD = 0.28) overall (JU: MP group compared to control group at 24 month follow-up), with a stronger weekday effect (35.34 CPM, SMD = 0.31).

**Table 3:** Effectiveness outcomes for average daily habitual behaviours

Outcome Measures	Intervention (*n* = 175)	Control (*n* = 155)	Difference (95% CI)	*p*-value	SMD
Average daily - habitual					
Adjusted MVPA (mean min/day)	64.80 (62.77, 66.83)	59.81 (57.78, 61.84)	**4.99 (1.01**,** 8.96)**	**0.014**	**0.29**
Adjusted ST (mean min/day)	417.50 (413.38, 421.62)	426.19 (422.07, 430.31)	**−8.69 (−16.76**,** −0.61)**	**0.035**	**−0.20**
Adjusted LPA (mean min/day)	296.88 (293.58, 300.18)	292.87 (289.57, 296.17)	4.00 (−2.46, 10.46)	0.225	0.14
Adjusted TPA (mean min/day)	361.07 (356.80, 365.34)	352.81 (348.54, 357.08)	8.26 (−0.11, 16.63)	0.053	0.22
Adjusted CPM (mean min/day)	611.32 (597.65, 624.99)	578.59 (564.92, 592.26)	**32.72 (5.93**,** 59.53)**	**0.017**	**0.28**
Weekday - average daily					
Adjusted MVPA (mean min/day)	66.17 (64.18, 68.16)	60.40 (58.41, 62.39)	**5.77 (1.87**,** 9.67)**	**0.004**	**0.35**
Adjusted ST (mean min/day)	428.22 (423.74, 432.70)	435.72 (431.24, 440.20)	−7.50 (−16.28, 1.28)	0.094	−0.2
Adjusted LPA (mean min/day)	295.65 (292.03, 299.27)	293.61 (289.99, 297.23)	2.04 (−5.06, 9.14)	0.574	0.06
Adjusted TPA (mean min/day)	361.24 (356.80, 365.68)	354.09 (349.65, 358.53)	7.15 (−1.55, 15.85)	0.107	0.18
Adjusted CPM (mean min/day)	607.52 (594.27, 620.77)	572.18 (558.93, 585.43)	**35.34 (9.38**,** 61.305)**	**0.008**	**0.31**
Weekend Day - average daily					
Adjusted MVPA (mean min/day)	59.43 (55.98, 62.88)	55.09 (51.64, 58.54)	4.18 (−2.53, 10.99)	0.222	0.15
Adjusted ST (mean min/day)	372.44 (366.00, 378.88)	393.91 (387.47, 400.35)	**−21.47 (−34.09**,** −8.85)**	**0.001**	**−0.4**
Adjusted LPA (mean min/day)	289.24 (284.37, 294.11)	277.49 (272.62, 282.36)	**11.75 (2.21**,** 21.29)**	**0.016**	**0.29**
Adjusted TPA (mean min/day)	349.10 (342.43, 355.77)	332.65 (325.98, 339.32)	**16.44 (3.37**,** 329.53)**	**0.014**	**0.29**
Adjusted CPM (mean min/day)	589.51 (564.99, 614.03)	570.90 (546.38, 595.42)	18.61 (−29.53, 66.575)	0.449	0.10

Adjusted for sex, ethnicity, baseline values, wear time, BMI, age, receipt of free school dinners, school and neighbourhood

*MVPA *moderate-to-vigorous physical activity, *ST *sedentary time, *LPA *light physical activity, *TPA *total physical activity, *CPM *counts per minute

*SMD *Standardised mean difference – Small effect – 0.2 to 0.5, Medium effect 0.5 to 0.8, Large effect ≥ 0.8

### Sensitivity analyses

The intervention effect on mean daily MVPA was robust to adjustment for attrition (Supplementary Table S3). The primary completers’ model estimated a mean difference of + 4.99 min/day (95% CI 1.01 to 8.96, *p* = 0.014). Re-analysis using robust standard errors produced a similar estimate (+ 4.63 min/day, 95% CI 1.60 to 7.65, *p* = 0.003). Inverse-probability weighting gave a nearly identical result (+ 4.48 min/day, 95% CI 1.01 to 7.96, *p* = 0.011). An augmented IPW model including baseline MVPA and YAP yielded a slightly smaller but still significant effect (+ 2.95 min/day, 95% CI 0.52 to 5.37, *p* = 0.017). These consistent findings indicate that missing data or selective follow-up are unlikely to have materially biased the results. Sensitivity analyses comparing completers, robust, and inverse-probability-weighted models are summarised in Supplementary Table S4.

### Secondary outcomes

Secondary outcomes (Table 4) (BMI z-scores, social emotional and behavioral health, and quality-of-life measures) had small, unimportant differences between groups. Exploratory analysis found evidence of meaningful effects from the JU: MP intervention for boys and South Asian children, with MVPA improving for both compared to the control group (+ 7.34 min/days, SMD = 0.36 and + 7.20 min/day, SMD = 0.52 respectively), there were smaller increases for girls and White British children.

**Table 4:** Effectiveness outcomes for anthropometry, strengths and difficulties and quality of life

Secondary Outcome Measures	Intervention (95% CI)	Control (95% CI)	Difference (95% CI)	*p*-value	SMD
Anthropometric					
Body Mass Index Z score (*n* = 1,103)	0.492 (0.43, 0.55)	0.559 (0.49, 0.22)	− 0.066 (−0.152, 0.0,211)	0.138	0.09
Waist Circumference inches (*n* = 1,098)	64.08 (63.65, 64.52)	63.40 (62.69, 64.12)	0.68 (−0.11, 1.48)	0.092	0.10
Social, emotional and behaviour difficulties Teacher Reported SDQ					
Total difficulties score (*n* = 675)	6.60 (5.08, 7.06)	6.70 (5.77, 7.63)	−0.634 (−1.99, 0.73)	0.362	−0.11
Emotional score (*n* = 676)	1.60 (1.34, 1.86)	1.76 (1.50, 2.01)	−0.157 (−0.519, 0.205)	0.395	−0.08
Conduct score (*n* = 677)	0.84 (0.63, 1.04)	1.03 (0.82, 1.24)	−0.192 (−0.486, 0.100)	0.199	−0.13
Hyperactivity score (*n* = 677)	2.69 (2.27, 3.10)	2.78 (2.39, 3.18)	−0.098 (−0.67, 0.478)	0.738	−0.04
Peer-problem score (*n* = 677)	0.95 (0.71, 1.19)	1.23 (1.01, 1.46)	−0.29 (−0.617, 0.042)	0.087	−0.19
Prosocial score (*n* = 677)	8.09 (7.71, 8.48)	8.34 (7.99, 8.70)	−0.248 (−0.768, 0.272)	0.350	−0.13
Quality of Life – Parent reported					
PEDSQL Score (*n* = 479)	80.96 (78.40, 83.52)	79.84 (77.38, 82.31)	1.12 (−2.44, 4.68)	0.538	0.08
Equation 5D Score (*n* = 700)	0.912 (0.90, 0.93)	0.915 (0.90, 0.93)	−0.003 (−0.267, 0.020)	0.783	−0.03

Adjusted for sex, ethnicity, baseline values, wear time, BMI, age, receipt of free school dinners, school and neighbourhood

*SMD *Standardised mean difference – Small effect – 0.2 to 0.5, Medium effect 0.5 to 0.8, Large effect ≥ 0.8

## Discussion

We report the results of a non-randomisd controlled trial of a whole system approach to improve population-level physical activity that reached over 30,000 children aged five to 14 years from a multi-ethnic and economically deprived area. The JU: MP intervention effectively mitigated the age-related decline in MVPA levels, resulting in a relative improvement of five minutes per day compared to the control group after 24 months. The study also found evidence of meaningful differences in average daily total physical activity, counts per minute and sedentary time between the JU: MP intervention and control group demonstrating effects of the intervention across multiple movement behaviours. The difference in overall average MVPA was driven by more substantial improvements on weekdays compared to weekends. On weekends, children in the JU: MP group improved their levels of light physical activity while also reducing sedentary time. While these effects were small (SMD 0.26 to 0.40), at a population level, this demonstrates the substantial public health impact of the JU: MP whole system approach to improve physical activity behaviours within multi-ethnic and socially and economically deprived communities.

Our study found that daily MVPA increased by an average of five minutes, with a noticeable effect size of 0.29, rising to 0.34 on weekdays. Compared to Metcalf et al.‘s meta-analysis of children’s physical activity interventions, which reported an effect size (SMD) of 0.16 and approximately four additional minutes of MVPA, our results are promising [58]. Specifically, the JU: MP whole system intervention which targets entire child populations (rather than just those with overweight or obesity), shows higher effectiveness than single or multi-component interventions (SMD 0.07 in Metcalf et al. [58]. Our findings also align with another recent meta-analysis [59], indicating a pooled effect size (SMD 0.34) for physical activity interventions among low-income and ethnic minority children. Notably, these interventions tend to be effective over shorter durations (< 13 weeks) but struggle to maintain long-term effects, highlighting the JU: MP intervention’s success in fostering behaviour change over two years. Unlike many studies, JU: MP operated as a comprehensive intervention implemented at scale and mitigated common pitfalls such as reduced MVPA levels observed during scaling due to implementation challenges like programme fidelity and adaptability [60]. JU: MP’s model included learning cycles, facilitating adjustments to these challenges, potentially overcoming issues encountered in other efficacy or effectiveness studies influenced by external factors like local partnerships, political support, and resource allocation.

Larger improvements in MPVA were observed on weekdays (+ 5.77 min/day), compared to weekends (+ 4.18 min/day). Explanations may be that this is when the children were exposed to physical activity opportunities in schools. Additionally, a large proportion of the children also attended Madrasas on weekdays due to their Muslim faith and the JU: MP intervention may have increased their physical activity opportunities in these settings too. Our additional research studies have evidenced organisational and cultural change for physical activity, which, in turn, has likely resulted in improved physical activity opportunities for pupils within these settings [61, 62]. Conversely, the overall differences in total physical activity and sedentary time were driven by improvements at the weekend (+ 17.2 min and − 22.15 min difference between groups respectively) suggesting that the workstreams operating at the ‘environment, community and family levels’ where physical activity is less structured were likely responsible for total physical activity and sedentary time improvements. The improvement in weekend TPA was predominantly due to improved LPA (+ 12.1 minutes difference between groups) which may reflect children swapping ST for light physical activities.

Evidenced by a previous meta-synthesis [63], it is important to understand potential sub-population effects of interventions to ensure equitable benefit and that interventions reach those with greatest health inequality[64]. The exploratory analysis found JU: MP had a greater effect on MVPA for boys (+ 7.3 min, SMD 0.36) than girls, and South Asian children (+ 7.2 min, SMD 0.52) than white British children. This indicates that the intervention components activated boys and South Asian children more than girls and White British children. The outcome for South Asian children is positive, given the need to address their lower levels of physical activity compared to White British peers [64]. Yet the challenge persists in improving girls’ physical activity. The lack of effect on secondary outcomes suggests that the intervention was not sufficient to change these outcomes within the follow-up period.

The findings demonstrate the value of taking a whole systems approach to improving population levels of physical activity. We have some initial ideas about what the important elements of the intervention might be, based on existing literature. These are: first, the JU: MP intervention had ‘a high use of system approaches’ (according to the analytical framework of Koorts et al.[12] and activated multiple systems, providing a broad array of physical activities with partners within neighbourhoods and across the Braford city district [65]. Second, it was underpinned by theory (complex adaptive systems thinking, the socio-ecological [66] and COM-B behaviour change models [67]) and previous research suggests that theory-based interventions tend to be more effective than those not referencing theory [68]. Third, the intervention and its individual elements were co-produced with stakeholders, perhaps enhancing the relevance, engagement, and sustainability of the intervention [69]. Fourth, a new district-wide physical activity strategy was co-developed with city-wide partners; we have clear evidence to demonstrate that this helped increase and strengthen relationships between organisations [70]. Fifth, national level advocacy and funding (via Sport England) ensured political support for a whole system place-sensitive way of working, with funding to support the development, delivery and evaluation of the intervention [14]. Sixth, the iterative learning process embedded within the intervention delivery allowed for continuing adjustments to the intervention and individual elements which may have met practical challenges of implementing interventions in practice and to adapt the intervention to contextual changes (e.g. social, political, economic and research evidence) [71]. Alongside the trial, our research group has also conducted a systematic and rigorous process evaluation which will help to unpick whether these intervention elements were important and further explore the mechanism of effects [17, 19].

The findings from this study are relevant to public health particularly because there is currently limited evidence for the impact of adopting a systems approach in physical activity interventions [11]. This study found that the JU: MP intervention improved MVPA by 5 min/day; given the dose-response relationship between physical activity and health outcomes for children and young people, any improvement in physical activity is better than none [65]. Moreover, the intervention slowed the rate of age-related decline in physical activity (equivalent to ~ 1.6 min/day/year) compared to controls (equivalent to ~ 3.7 min/day/year), even more important given that the JU: MP intervention group had higher levels of deprivation than the control group. This compares favourably to UK and European cohort studies that have shown children’s physical activity levels decline from the age of six to seven through to fourteen to fifteen years at a rate of 2.2 min/day/year [72] and ~ 2.8 min/day/year (3) respectively. As physical activity behaviours in mid-childhood track into adolescence (6), if the effects of JUMP are sustained year-on-year, these could amount to a difference of ~ 12 min of physical activity per day by the age of fourteen to fifteen. The threshold daily time for MVPA to be health enhancing for children is not clearly defined and is thought to be between 30 and 60 min/day on 3 or more days a week [73]. Thus, a possible 12-minute improvement by adolescence at a population level (if behaviours are sustained year on year) could shift a significant number of young people out of the ‘inactive, < 30 minutes/day’ zone and into the > 30 min zone, and achieve important population level public health improvements. Further research would be required to track the young people and assess long-term intervention impact in mid-adolescence to validate these claims.

There is a paucity of detailed descriptions of the operationalisation of whole system public health interventions. JU: MP provides a clear example of an effective whole system approach, with resources detailing the operationalisation of the intervention that local and national governments can learn from [65]; as such this research can be viewed as an early step in the development and adoption of effective whole system approaches to physical activity. However, complex whole system interventions cannot simply be transplanted into new localities, decision-makers need to consider the appropriateness of both the intervention components themselves (e.g. the workstreams and their interactions) and how the intervention might interact with the context in which it is being implemented. For researchers, this means evaluations need to be appropriate and multifaceted. Recently released guidance and framework [71, 74] suggest that although research should still explore the effectiveness of interventions, a broader range of questions should also be posed, e.g., what are the wider impacts? What is the value relative to the resources to deliver? How does it work, for who and why? considering the context in which it is implemented. How does it contribute to system change? The evaluation of JU: MP has taken this multi-faceted approach and has used a mixed methods process evaluation which will explore these questions. Future focuses of research on the JU: MP intervention include, how can the JU: MP intervention be sustained locally, how can the intervention be scaled out to the whole of the Bradford District, what is the impact of this and what is the long-term impact of the intervention upon physical activity and health outcomes? Whether the JU: MP intervention effects can be sustained over the longer-term with lower resources? The trial is undertaking a 36-month follow-up which will further explore this alongside a cost-benefit evaluation of the intervention.

This study has several strengths. To our knowledge it represents the first published effectiveness evaluation of a whole systems approach to increasing children’s physical activity, demonstrating its feasibility despite significant time and resource demands. A pragmatic intention-to-treat design ensured all eligible children within recruited schools were included in the analysis, regardless of their engagement in the JU: MP intervention, thus assessing real-world effectiveness. The use of accelerometry to measure physical activity is a key strength, providing robust and reliable outcome data. The study’s non-randomised controlled trial design whilst introducing some bias into the study, allowed for naturalistic evaluation in underserved settings, with matched control neighbourhoods improving comparability. Furthermore, the focus on deprived and ethnically diverse neighbourhoods highlights the study’s relevance for addressing health inequalities, and populations often underrepresented in physical activity interventions.

Limitations include the non-randomised study design; which, while pragmatic and in line with recent guidance on natural experimental evaluations [75], restricts causal inference due to the absence of randomisation and potential unmeasured confounding. In designing the trial, an ICC of 0.01 was assumed, derived empirically from the JU: MP pilot (unpublished), which estimated a ICC of 0.007 for accelerometer-measured MVPA. This value was conservatively rounded up to 0.01 to ensure adequate power. Although more recent studies have reported higher ICCs for school-based accelerometer MVPA in England (e.g., 0.04–0.08 [44]), the lower ICC observed in the JU: MP pilot is likely due to the study’s broader neighbourhood-level sampling frame and the substantial within-school heterogeneity in physical activity typical of multi-ethnic, deprived urban settings. Previous work has shown that ICCs for physical activity can vary widely depending on population diversity, school size, and contextual factors such as deprivation and built environment [44, 76]. These considerations support the plausibility of our design assumption and indicate that the analytic models appropriately accounted for clustering. Furthermore, the sensitivity analyses confirmed that the intervention effect remained robust across completers, robust-standard-error, and weighted models, suggesting the main findings are unlikely to be materially biased by design assumptions or missing data. Additionally, individual exposure to the various elements of the JU: MP intervention was not assessed, complicating efforts to isolate specific components driving the observed effects. Furthermore, the geographical focus on underserved areas in Bradford and Yorkshire may limit the generalisability of findings to other populations or regions. Furthermore, only two measurement points, baseline and 24 months, were included, which may have missed short-term impacts or changes. Data completeness was another limitation, with only 22.7% of children meeting accelerometer wear-time criteria at both time points, potentially affecting the interpretation and generalisability of findings. This aligns with previous studies, where accelerometer compliance rates are often lower in socioeconomically deprived populations [77–80]. Barriers such as participant burden, device discomfort, and lower parental engagement may have contributed to data loss [47, 79, 80]. Future studies should explore strategies to enhance compliance, including wrist-worn devices, reduced wear-time thresholds, and targeted support. A further limitation was the high level of missingness in several key covariates, particularly parent-reported questionnaire data, which limited the reliability of multiple imputation under a plausible missing‐at‐random assumption, therefore an intention-to-treat analysis as originally planned was not appropriate for this analysis. Consequently, we have presented a completers’ analysis for this paper while recommending future work with enhanced data completeness or targeted auxiliary measures to support a full intention-to-treat approach. Finally, the study was conducted during the COVID-19 pandemic (recruitment in 2021), which may have influenced physical activity levels and JU: MP intervention delivery. Furthermore, due to staffing shortages, illness, and travel restrictions during the ongoing COVID-19 pandemic, the trial was retrospectively registered (ISRCTN14332797), this was despite having received ethical approval and finalised protocol in July 2021. We acknowledge this is a limitation, as prospective registration enhances confidence in the validity of findings; however, large, community-based, multi-team studies can face capacity challenges, and although we, the authors are confident the retrospective registration has no impact on the results discussed in this paper, we are being transparent about the circumstances that led to this delay.

## Conclusion

The current study presents the findings of the first trial of a whole system, population-level physical activity intervention that reached over 30,000 children aged five to 14 years from a multi-ethnic and economically deprived area. The JU: MP intervention was found to be effective with levels of physical activity in the intervention group higher than the control, suggesting that this systems approach is a promising way of mitigating the age-related decline in moderate to vigorous physical activity in children. However, these results should be interpreted with caution given that only 22.7% of consented children provided valid accelerometer data and that neighbourhoods were not randomised. Future work should prioritise strategies to improve accelerometer wear-time, such as wrist-worn devices, reduced wear-time thresholds, and significantly enhance participant support, and, where feasible, adopt more rigorous designs (for example, cluster randomised controlled trial) to strengthen causal inference in whole-system evaluations. Nonetheless, this study offers evidence that whole systems approaches hold promise for addressing population levels of physical activity, whilst also tackling inequalities and improving public health outcomes at scale.

## Supplementary Information

Supplementary Material 1.

Supplementary Material 2.

Supplementary Material 3.

Supplementary Material 4.

Supplementary Material 5.

## Data Availability

The datasets used and/or analysed during the current study are available from the corresponding author on reasonable request.
