# Supplementary figures and images for: The effectiveness of JU:MP a whole system approach to improve physical activity of children aged 5 to 11 years living in multi-ethnic and socio-economically deprived communities: a non-randomised controlled trial
Source: BMC Public Health. 2025 Dec 7;26:152. doi: 10.1186/s12889-025-25772-9 (PMC12797546; doi:10.1186/s12889-025-25772-9)

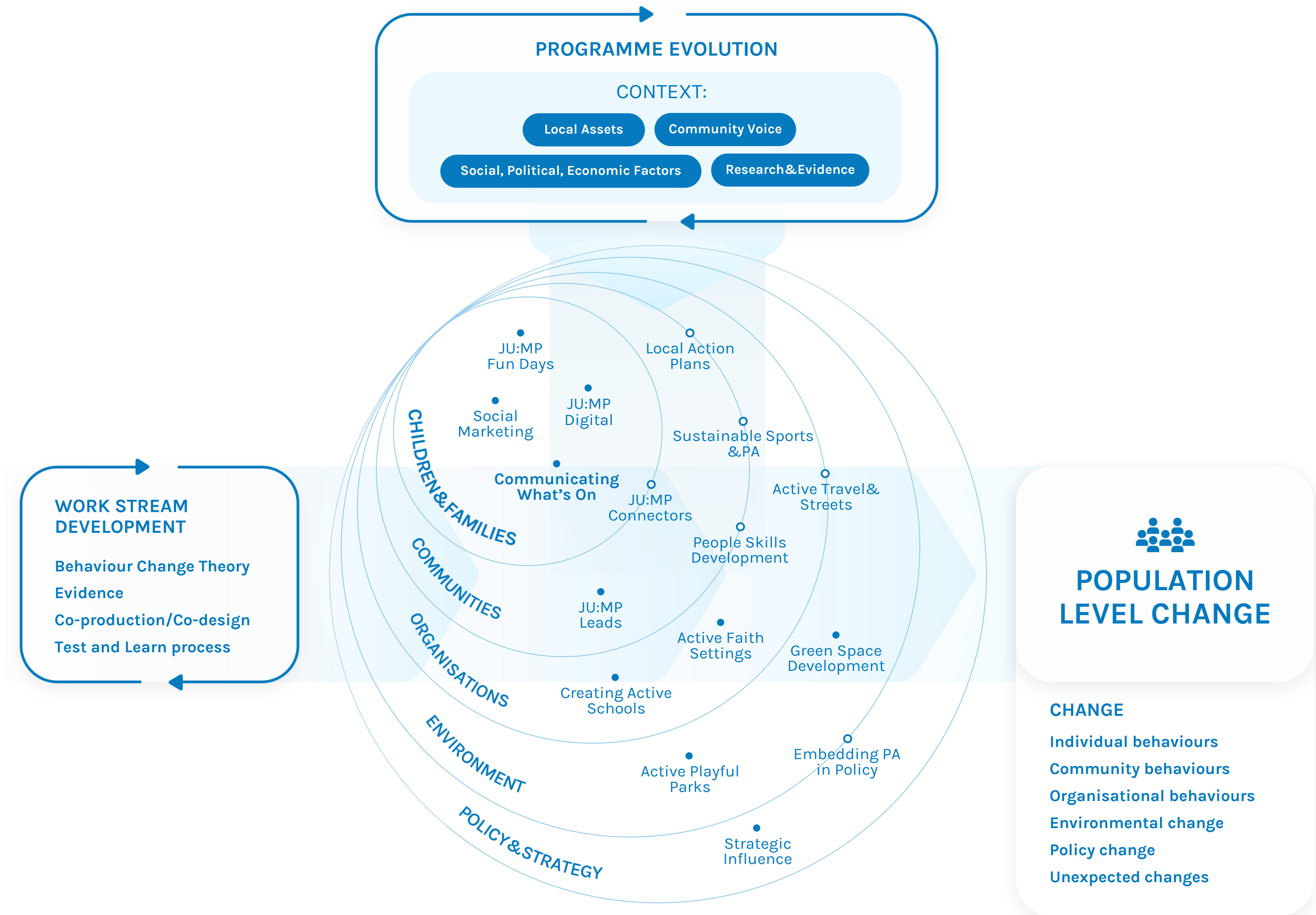

Supplement: Supplementary file 2 — Supplementary Material 2. [file 12889_2025_25772_MOESM2_ESM.pdf]

Supplement: Supplementary file 4 — Supplementary Material 4. [file 12889_2025_25772_MOESM4_ESM.docx]
