# Supplementary material for: The effectiveness of JU:MP a whole system approach to improve physical activity of children aged 5 to 11 years living in multi-ethnic and socio-economically deprived communities: a non-randomised controlled trial
Source: BMC Public Health. 2025 Dec 7;26:152. doi: 10.1186/s12889-025-25772-9 (PMC12797546; doi:10.1186/s12889-025-25772-9)
Supplement: Supplementary file 3 — Supplementary Material 3. [file 12889_2025_25772_MOESM3_ESM.docx]

**Supplementary Tables 1 to 4**

**Supplementary TS1:** Characteristics and descriptives of recruited schools and neighbourhoods

|  | No. Schools | No. pupils in Years 1 to 3 | Median % of pupils known to be eligible for free school meals | Median % of pupils classified as White British | Median % of pupils classified as South Asian | Median School IMD* decile | |
| --- | --- | --- | --- | --- | --- | --- | --- |
| Neighbourhood 1 |  |  |  |  |  |  | |
| Intervention | 6 | 789 | 25.9 | 73.15 | 10.45 | 2.5 | |
| Control | 7 | 718 | 34.9 | 70.9 | 9.15 | 2 | |
| Total | 13 | 1507 | 28.9 | 72.1 | 9.65 | 2 | |
| **Difference** | **-1** | **71** | **-9** | **2.25** | **1.3** | **0.5** | |
| Neighbourhood 2 |  |  |  |  |  |  | |
| Intervention | 5 | 840 | 30.6 | 0.8 | 86.2 | 1 | |
| Control | 7 | 798 | 22.1 | 3.2 | 85.5 | 2 | |
| Total | 12 | 1638 | 24 | 1.55 | 85.85 | 1.5 | |
| **Difference** | **-2** | **42** | **8.5** | **-2.4** | **0.7** | **-1** | |
| Neighbourhood 3 |  |  |  |  |  |  | |
| Intervention | 6 | 613 | 42.1 | 68.75 | 2.3 | 1.5 | |
| Control | 6 | 650 | 37 | 81.9 | 2.45 | 1.5 | |
| Total | 12 | 1263 | 42.1 | 80.5 | 2.3 | 1.5 | |
| **Difference** | **0** | **-37** | **5.1** | **-13.15** | **-0.15** | **0** | |
| Total |  |  |  |  |  |  | |
| Intervention | 17 | 2242 | 30.7 | 57.8 | 17.5 | 1 | |
| Control | 20 | 2166 | 26.6 | 61.35 | 17.9 | 2 | |
| Total | 37 | 4408 | 30 | 57.8 | 17.5 | 1 | |
| **Difference** | **-3** | **76** | **4.1** | **-3.55** | **-0.4** | **-1** | |
| Source of data was Department of Education Census Academic year 2021/22 - Schools, pupils and their characteristics  <https://explore-education-statistics.service.gov.uk/find-statistics/school-pupils-and-their-characteristics/2021-22>  * IMD = Index of Multiple Deprivation, based upon school postcode | | | | | | |  |

| **Statistic** | **Mean** | **SD** | **Min** | **Max** |
| --- | --- | --- | --- | --- |
| Stabilised weight | 1.02 | 0.14 | 0.84 | 1.32 |

**Supplementary Table S2.** Descriptive statistics of attrition weights (primary outcome: mean daily MVPA)

**Notes:** Stabilised inverse-probability-of-attrition weights were derived from a logistic regression predicting the probability of valid follow-up accelerometer data using baseline sex, ethnicity, trial arm, and free school meal eligibility. The narrow range and mean near 1.0 indicate well-behaved weights with no evidence of extreme values.

**Supplementary Table S3.** Mixed-effects regression model results for the primary outcome (mean daily MVPA, minutes/day) — Completers analysis

| **Variable** | **Coefficient** | **Std. Error (Bootstrap)** | **z** | **p-value** | **95% CI** |
| --- | --- | --- | --- | --- | --- |
| Trial condition (Intervention vs Control) | 4.99 | 2.03 | 2.46 | 0.014 | 1.01, 8.96 |
| Female | -7.52 | 1.99 | -3.78 | <0.001 | -11.42, -3.62 |
| Ethnicity: South Asian | -11.01 | 2.40 | -4.58 | <0.001 | -15.72, -6.30 |
| Ethnicity: Other | -3.32 | 3.29 | -1.01 | 0.313 | -9.76, 3.12 |
| Baseline MVPA | 0.50 | 0.06 | 8.69 | <0.001 | 0.39, 0.61 |
| Follow-up wear time | 0.04 | 0.02 | 1.82 | 0.069 | -0.00, 0.08 |
| BMI z-score | -1.83 | 0.73 | -2.52 | 0.012 | -3.25, -0.40 |
| Age (follow-up) | -3.64 | 1.09 | -3.33 | 0.001 | -5.79, -1.50 |
| Free school meal eligibility | -0.08 | 2.26 | -0.03 | 0.973 | -4.51, 4.36 |
| Constant | 39.30 | 18.48 | 2.13 | 0.033 | 3.08, 75.52 |

**Random effects:**

- Neighbourhood variance = 0.00 (95% CI: 0.00, 1.94)
- School variance = 3.87 (95% CI: 0.05, 288.34)
- Residual variance = 301.24 (95% CI: 248.03, 365.85)

**Notes:** Model includes random intercepts for school and neighbourhood. Model adjusted for sex, ethnicity, baseline MVPA, wear time, BMI z-score, age, and free school meal eligibility. *N = 327*.

**Supplementary Table S4.** Sensitivity analyses for the primary outcome (mean daily MVPA, minutes/day)

| **Model** | **Description** | **Mean Difference (95% CI)** | **p-value** |
| --- | --- | --- | --- |
| Completers model | Primary mixed-effects model with bootstrap SEs | **+4.99 (1.01, 8.96)** | **0.014** |
| Robust SE model | Mixed-effects model with robust standard errors | **+4.63 (1.60, 7.65)** | **0.003** |
| IPW model | Weighted mixed-effects model (sex, ethnicity, FSM, trial arm) | **+4.48 (1.01, 7.96)** | **0.011** |
| Augmented IPW model | Weighted model additionally adjusting for baseline MVPA and YAP | **+2.95 (0.52, 5.37)** | **0.017** |

**Notes:** All models include random intercepts for school and neighbourhood. IPW = inverse-probability weighting; FSM = free school meals; YAP = Youth Activity Profile. All analyses conducted in Stata v17.
