# Supplementary material for: The effectiveness of JU:MP a whole system approach to improve physical activity of children aged 5 to 11 years living in multi-ethnic and socio-economically deprived communities: a non-randomised controlled trial
Source: BMC Public Health. 2025 Dec 7;26:152. doi: 10.1186/s12889-025-25772-9 (PMC12797546; doi:10.1186/s12889-025-25772-9)
Supplement: Supplementary file 5 — Supplementary Material 5. [file 12889_2025_25772_MOESM5_ESM.pdf]

Permission to use the Strengths and Difficulties Questionnaire

From: Youthinmind <support@youthinmind.com>
Sent: Monday, November 17, 2025 09:01
To: Daniel Bingham <d.bingham@bradford.ac.uk>
Subject: Re: Request for Confirmation of Permission to Use the Teacher-Reported SDQ in a Non-Commercial Research Study

Caution External Email: Do not click any links or open any attachments unless you trust the sender and know that the content is safe.

Hi Daniel,

We give Dr Daniel Bingham, University of Bradford permission to publish the study titled *"The Effectiveness of JU:MP a Whole System Approach to Improve Physical Activity of Children Aged 5 to 11 Years Living in Multi-ethnic and Socio-economically Deprived Communities: A Non-Randomised Controlled Trial."* on the basis that Dr Daniel Bingham has confirmed that the official SDQ in PDF format was downloaded from the SDQ website without any modifications and was administered using the pen-and-paper method.

Please note that this confirmation is based on the statement made by Dr Daniel Bingham affirming his adherence to our copyright and licensing conditions, which we have not independently verified.

Best wishes,
Helena Hamilton
Youthinmind
